# Extracellular matrix: unlocking new avenues in cancer treatment

**DOI:** 10.1186/s40364-025-00757-3

**Published:** 2025-05-27

**Authors:** Jia Jing Lee, Khuen Yen Ng, Athirah Bakhtiar

**Affiliations:** School of Pharmacy, Monash University Malaysia, Jalan Lagoon Selatan, Bandar Sunway, 47500 Selangor Malaysia

**Keywords:** Extracellular matrix, Cancer, Cancer resistance, Tumor microenvironment

## Abstract

The extracellular matrix (ECM) plays a critical role in cancer progression by influencing tumor growth, invasion, and metastasis. This review explores the emerging therapeutic strategies that target the ECM as a novel approach in cancer treatment. By disrupting the structural and biochemical interactions within the tumor microenvironment, ECM-targeted therapies aim to inhibit cancer progression and overcome therapeutic resistance. We examine the current state of ECM research, focusing on key components such as collagen, laminin, fibronectin, periostin, and hyaluronic acid, and their roles in tumor biology. Additionally, we discuss the challenges associated with ECM-targeted therapies, including drug delivery, specificity, and potential side effects, while highlighting recent advancements and future directions. This review underscores the potential of ECM-focused strategies to enhance the efficacy of existing treatments and contribute to more effective cancer therapies.

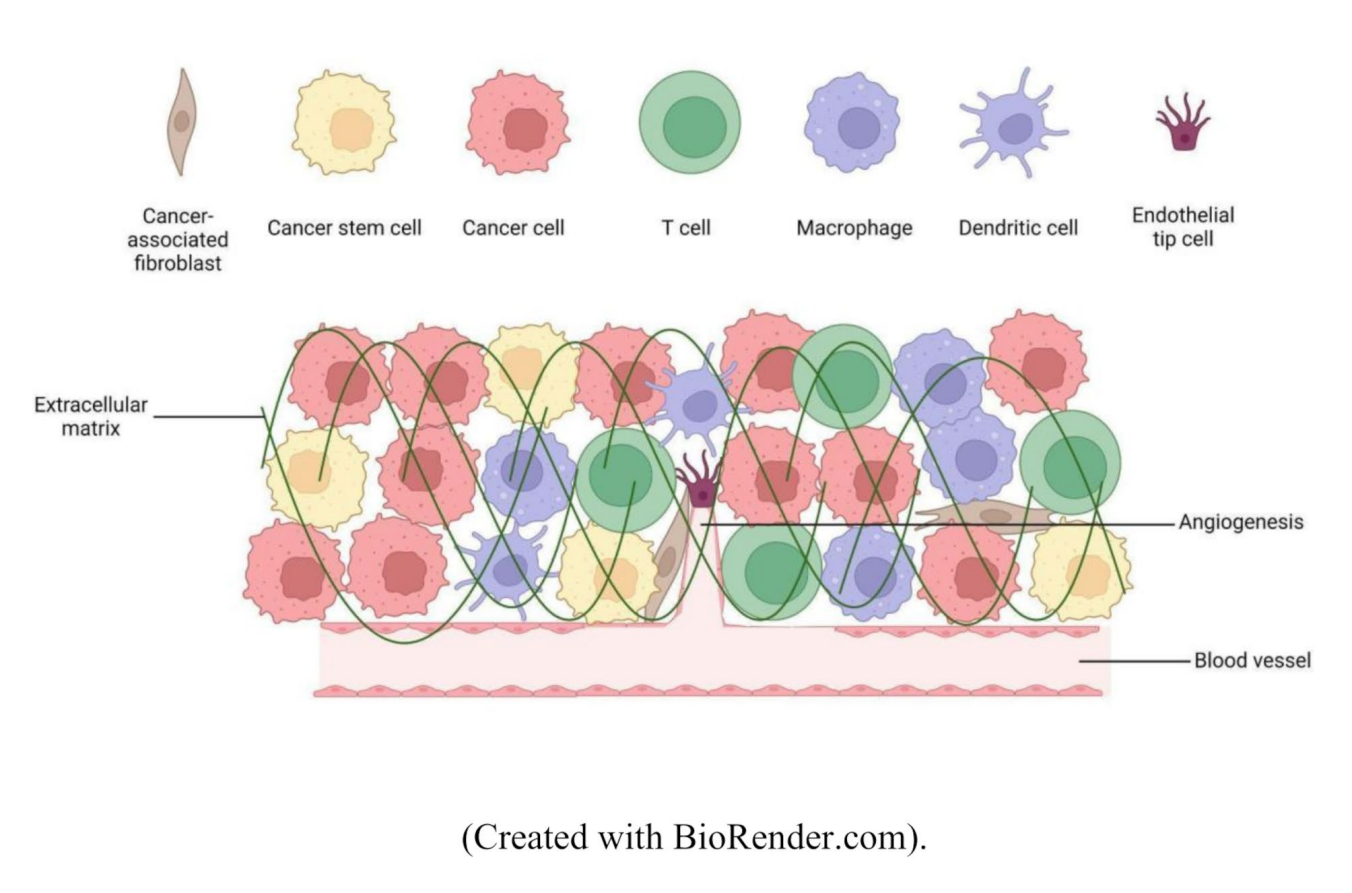

## Introduction

**Fig. 1:**
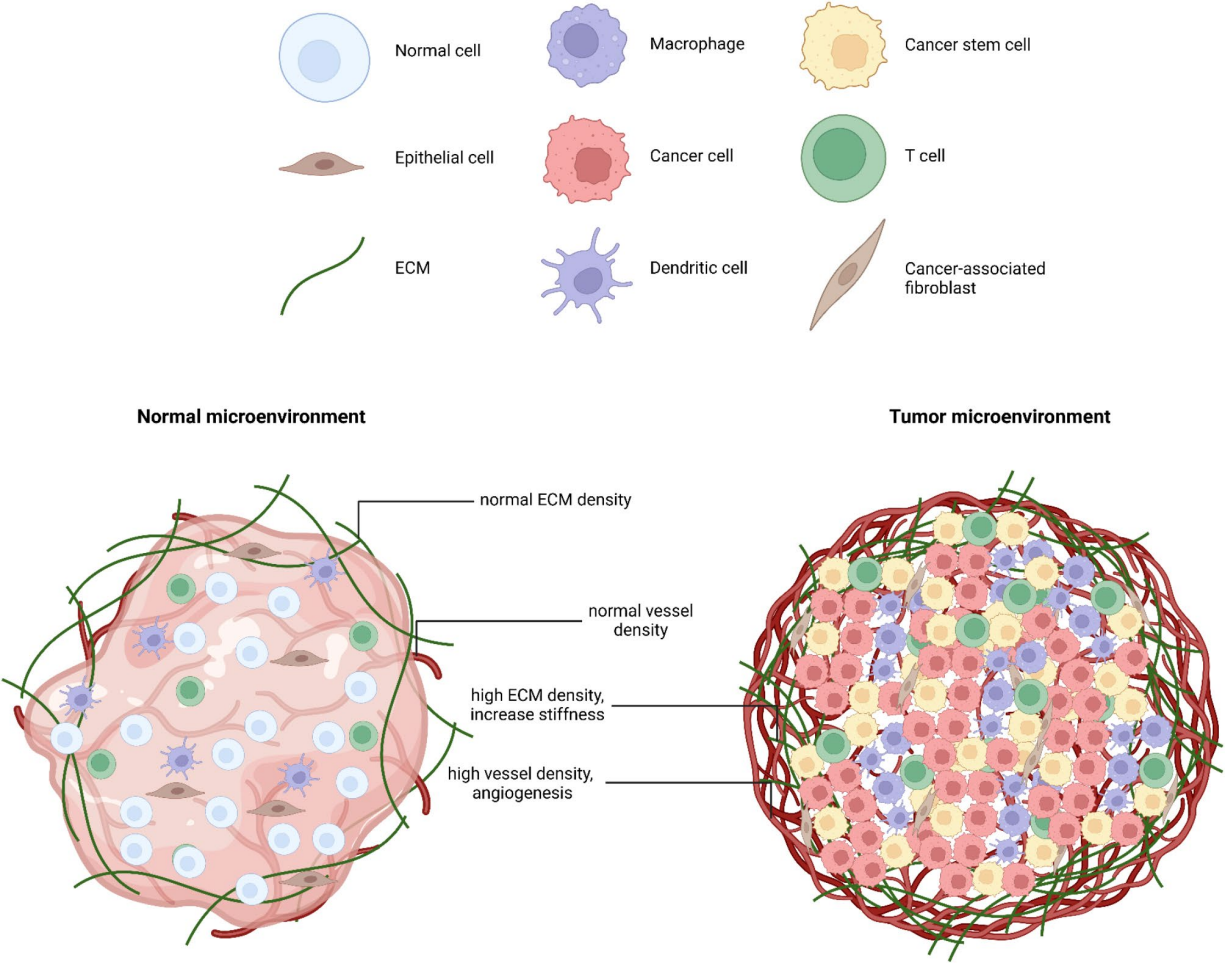
A comparison of the ECM and vessel density between the normal microenvironment and the TME highlights significant differences driving cancer progression. Angiogenesis, the formation of new capillaries, supplies tumors with the blood necessary for their survival and growth. As a structural component of the TME, the ECM plays an essential role in tumor development and progression. The transition from a normal microenvironment to a TME involves several key changes, including an altered cellular composition marked by increased proliferation of cancer cells, cancer-associated fibroblasts, and reprogrammed immune cells. The TME becomes more degraded, facilitating tumor invasion, while abnormal angiogenesis results in poorly structured blood vessels, leading to hypoxia and acidosis. These conditions, combined with altered metabolic processes like the Warburg effect, support rapid tumor growth. Furthermore, immune cells within the TME are often suppressed, enabling tumors to evade immune surveillance. Chronic inflammation within the TME further accelerates tumor progression. (Created with BioRender.com)

The tumor microenvironment (TME) refers to the intricate surroundings of a tumor, consisting of various non-cancerous cells, molecules, and structures that interact with and influence cancer growth and progression (Fig. 1). The TME includes cancer-associated fibroblasts (CAFs), immune cells, endothelial cells, and mesenchymal stem cells (MSCs), all of which play critical roles in tumor development. These roles encompass supporting tumor growth, suppressing immune responses, promoting blood vessel formation, and facilitating cancer cell migration [1–3].

The extracellular matrix (ECM), which offers structural support to cells [4], plays a dual role within the TME, acting as both an inhibitor and a promoter of tumorigenesis. Under normal conditions, components of the ECM, such as type IV collagen within the basement membrane, maintain tissue homeostasis and inhibit cancer cell migration by forming a physical barrier and providing biochemical signals that suppress invasive behavior. Type IV collagen also contributes to the structural integrity of the basement membrane, limiting tumor cells’ access to surrounding tissues [5]. However, during tumor progression, the ECM undergoes frequent remodeling, including the degradation of type IV collagen by matrix metalloproteinases (MMPs). This degradation compromises the basement membrane, facilitating tumor cell invasion and metastasis [6].

The rapid growth of tumors often outspaces their oxygen supply, creating hypoxic (low-oxygen) regions. In response, cancer cells activate survival pathways and promote angiogenesis to enhance their blood supply [7–10]. Signaling molecules, such as cytokines, chemokines, and growth factors are abundant in the TME, facilitating communication between cancer cells and their microenvironment. These molecules often contribute to immune suppression and tumor growth [2, 11]. For example, immune-suppressing cells such as tumor-associated macrophages (TAMs) and regulatory T cells (Tregs) can be recruited to the TME, enabling the tumor to evade immune surveillance [12]. Metabolic alterations, such as the Warburg effect, where cancer cells predominantly rely on glycolysis even in the presence of oxygen - further influence the behavior of both tumor and stromal cells [13].

Given its pivotal role in supporting tumor survival, invasion, metastasis, and therapy resistance, the TME represents a promising target for novel therapeutic strategies [14]. Approaches that target the ECM within the TME are particularly compelling, as they can disrupt the mechanical properties that promote tumor progression. By interfering with ECM components, these therapies may inhibit tumor cell migration, alter tumor stiffness, and prevent metastasis. Alongside ECM-targeting therapies, other strategies-such as immunotherapy and anti-angiogenic agents- seek to counteract the tumor-supportive functions of the TME.

Advancing our understanding of the complex interactions within the TME holds significant potential for developing more effective therapies, overcoming drug resistance, and improving patient outcomes [15–16].

## Extracellular matrix

The TME is a complex ecosystem surrounding a tumor, composed primarily of various non-cancerous cells, signaling molecules and the ECM. The ECM provides structural and biochemical support to tissues and is continuously deposited, remodeled, and degraded to maintain tissue homeostasis. However, in cancer, extensive reorganization of the ECM becomes a prerequisite for tumor development and progression. This has prompted the exploration of targeting the ECM as a therapeutic strategy, offering a novel approach to disrupting tumor growth by specifically modulating ECM components.

The ECM is a dynamic network of proteins and carbohydrates that not only provides structural support but also actively regulates cell behavior. Table 1 offers a concise summary of its main components—including collagens, elastin, proteoglycans, glycoproteins, and matricellular proteins—detailing their unique properties, functions, and clinical relevance. This overview lays the foundation for a deeper discussion on how alterations in these components drive ECM remodeling and influence tumor progression.

**Table 1:** Role of extracellular matrix components in cancer and their clinical implications [1, 4, 6]

ECM Component	Molecules	Key Properties	Main Functions	Clinical Implications
Collagens	COL I, II, III, IV, V	Structural support, ECM stiffness	Increase rigidity, promotes tumor progression and metastasis	Tumor progression and metastasis
Laminin	LN332, LN 511 (LAMA5, LAMB1, LAMC1), LN332 (LAMA3, LAMB3, LAMC2), LAMA1	Cell adhesion, migration, differentiation, self-polymerization, confers resistance to apoptosis	Binds to integrins to regulate tumor growth and invasion, enhances tumor cell attachment	High LN332 levels are associated with aggressive cancers, immune evasion and therapy resistance
Fibronectin	Cellular FN, plasma FN, FN isoforms (EDA-FN, EDB-FN)	Cell adhesion, migration, proliferation, apoptosis	Binds integrins (α5β1, αvβ3) to promote cancer cell attachment	Tumorigenesis, mechanical signal transduction into biochemical cues, potential biomarker for aggressive cancers
Periostin	-	Tissue repair, collagen-rich connective tissues development	Binds with integrins to influence cell migration and regulates collagen fibrillogenesis	Tumor progression, affect the size and quantity of metastatic lesions
Hyaluronic Acid	-	Proliferation, migration, invasion	Interacts with CD44 and RHAMM to cause inflammation and early tumor formation	Tumor growth, invasion and drug resistance

## The role of ECM in the TME

The TME is a dynamic structure comprising multiple cell types embedded within a remodeled ECM. This interaction enables bidirectional communication between cells and ECM macromolecules through both (i) direct cell-cell or cell-free contacts and (ii) soluble factors and vesicles that facilitate communication [15], influencing tumor progression and metastasis [16–20]. Mediators of these interactions include secreted molecules (e.g., chemokines, cytokines, and growth factors) and extracellular vesicles, which enable the horizontal transfer of genetic or biomaterial information [15]. Tumor cells exploit the cellular and non-cellular components of the TME to support their growth and survival in hostile conditions. These mediators include soluble factors and materials like cell-free DNA (cfDNA), apoptotic bodies, circulating tumor cells (CTCs), and exosomes [21].

The ECM plays a pivotal role in regulating cell behavior and is primarily secreted by CAFs, which produce more ECM proteins than normal fibroblasts. CAFs, the dominant ECM producers within the TME, are activated in response to signals from cancer cells and the surrounding stromal elements, leading to excessive ECM production [22, 23]. Proteins such as collagen, fibronectin and proteoglycans [24, 25] are overproduced, contributing to increased ECM rigidity and altered architecture, which in turn facilitate cancer cell migration, invasion and metastasis [26].

Additionally, the ECM influences cell signaling [27], enhances tumor cell survival [28], and supports angiogenesis [29]. Modulated cellular signals can induce tumor cell dormancy by creating niches where cancer cells remain quiescent and shielded from therapeutic agents. Dormant cells pose a significant challenge, as they can later reactivate and drive metastasis [30]. ECM remodeling within the TME- such as changes in stiffness and composition-further drives immune evasion, angiogenesis and epithelial-mesenchymal transition (EMT), all of which contribute to tumor aggressiveness. Targeting the intricate interplay between the TME and ECM is a promising therapeutic strategy to inhibit cancer progression and prevent relapse [31].

These characteristics make CAFs and the ECM critical therapeutic targets for disrupting the tumor-supportive microenvironment. Beyond CAFs, other cell types, including macrophages, cancer cells, and endothelial cells, also contribute to ECM remodeling [32]. TAMs secrete ECM proteins and growth factors that remodel the TME [33–35], while cancer cells produce ECM components that promote their own survival and migration, facilitating tumor progression and metastasis [4, 36, 37]. Endothelial cells contribute laminin [38, 39] and collagen [40–42] to the ECM, which are essential for angiogenesis and the formation of new blood vessels [43, 44].

## ECM as a modulator of tumor progression

The ECM not only provides structural support for cells but also acts as a reservoir for growth factors and cytokines, regulating their availability within the TME [45, 46]. ECM components, such as proteoglycans and glycosaminoglycans, bind to growth factors like VEGF, EGF, and TGF-β, controlling their release during tissue remodeling or cell signaling events. This controlled release influences various cellular processes, including cell proliferation, migration, and survival [47–50], thereby promoting tumor growth, angiogenesis and metastasis [6, 51, 52].

Tissues and organs possess distinct ECM compositions, shaped by variations in the expression of ECM genes, alternative splicing and post-translational modifications [53, 54]. Under normal conditions, the ECM undergoes continuous renewal as part of tissue development and homeostasis. This dynamic interaction allows ECM to influence adhesion, proliferation, migration, and survival while cells remodel the ECM to support these processes [55]. Tissue homeostasis is maintained by balancing ECM synthesis and degradation [56–58]. However, in cancer, this balance is disrupted. Tumor and stromal cells secrete enzymes like MMPs to degrade ECM components while simultaneously producing excessive ECM proteins, leading to increased tissue stiffness and altered architecture [4, 6, 59]. This persistent remodeling promotes tumor progression by enhancing cell migration, angiogenesis, and immune evasion [4, 58, 60].

Emerging evidence highlights the significant role of the ECM in disease progression and chemoresistance [61]. While cells produce the ECM, it serves as the environment where cells and biomolecules interact, influencing cellular behavior. Cellular functions and phenotypes depend on both gene expression and ECM-derived signals, suggesting that targeting the ECM may be an effective cancer treatment strategy [6, 62].

## Desmoplasia and ECM stiffness

The ECM becomes stiffer in cancer due to increased collagen and hyaluronic acid accumulation. Persistent tissue injury leads to excessive ECM protein production, resulting in a fibrotic state known as desmoplasia [63]. Studies have linked increased ECM deposition with tumor initiation and growth [43, 44].

Desmoplasia, a hallmark of ECM remodeling in cancer, is characterized by excessive ECM deposition and increased rigidity. Elevated ECM stiffness alters the mechanical properties of the TME [36, 64, 65], activating mechanotransduction pathways through integrins and focal adhesions. These pathways promote cancer cell proliferation, migration, and invasion [65, 66]. Increased ECM stiffness disrupts normal tissue architecture, recruits stromal cells like CAFs, and exacerbates fibrosis, creating a pro-tumorigenic niche [67]. These biophysical changes enhance angiogenesis and limit immune cell infiltration [68], fostering an environment that supports tumor progression and therapy resistance.

Tumor cells exploit ECM stiffness to facilitate migration through a process called durotaxis [69], whereby cells sense and move toward stiffer ECM regions, contributing to metastasis [70]. Research indicates that circulating tumor cells preferentially migrate to and settle in tissues and organs with higher ECM production [71], resulting in greater ECM stiffness. This stiffer ECM provides a favorable microenvironment for tumor cells, promoting invasive behavior and facilitating the establishment of secondary tumors [72].

## Targeting the ECM as a therapeutic strategy

The unique properties of the ECM within the TME make it an appealing target for cancer therapy. By specifically targeting specific ECM components, it may be possible to enhance the distribution and effectiveness of treatments [73]. Such therapies could disrupt tumor progression, inhibit angiogenesis, and improve drug delivery, as the dense ECM often acts as a barrier to treatment [73, 74]. During the initial stages of tumor development, stromal cells produce high levels of ECM proteins to shield healthy tissue from cancerous cells, leading to tissue hardening around the tumor [58]. ECM-targeted therapies could also reduce fibrosis by limiting excessive ECM deposition, potentially reversing fibrosis and improving organ function [75–77]. Furthermore, ECM modulation could support tissue repair and promote stem cell differentiation, offering potential applications in regenerative medicine [78–80].

## Challenges in targeting the ECM

Despite its therapeutic potential, targeting the ECM presents significant challenges. The ECM’s complexity and heterogeneity, which vary across tissue types and tumor subtypes, complicate the development of selective therapies [72]. Under normal physiological conditions, ECM remodeling is a tightly regulated process involving numerous proteins with distinct roles in maintaining homeostasis [75]. During tumorigenesis, ECM remodeling can both promote and inhibit tumorigenesis [4, 26, 81, 82]. This dual role complicates therapeutic approaches, as the ECM functions as both a tumor supressor and a promoter [83]. Tumors may also adapt to ECM-targeting treatments by upregulating alternative ECM components or activating compensatory survival pathways [84].

Paradoxically, some ECM-targeting therapies may inadvertently promote tumor progression by releasing growth factors or creating conditions favorable to invasion and metastasis [85]. Off-target effects on normal tissues pose another challenge, as the ECM plays a critical role in preserving tissue homeostasis [86]. Disrupting ECM integrity could lead to adverse outcomes such as impaired wound healing [87, 88] or organ dysfunction [89, 90].

Efforts to remodel the ECM to improve drug delivery [91] may also enhance tumor cell invasiveness [92]. The lack of specific biomarkers to identify patients likely to benefit from ECM-targeting therapies further limits their effectiveness. Additionally, some ECM-modulating approaches may suppress immune responses, potentially reducing the efficacy of combined treatments such as immunotherapy [6, 36, 93].

## Targeting ECM components as a cancer treatment approach

### Collagens

**Fig. 2:**
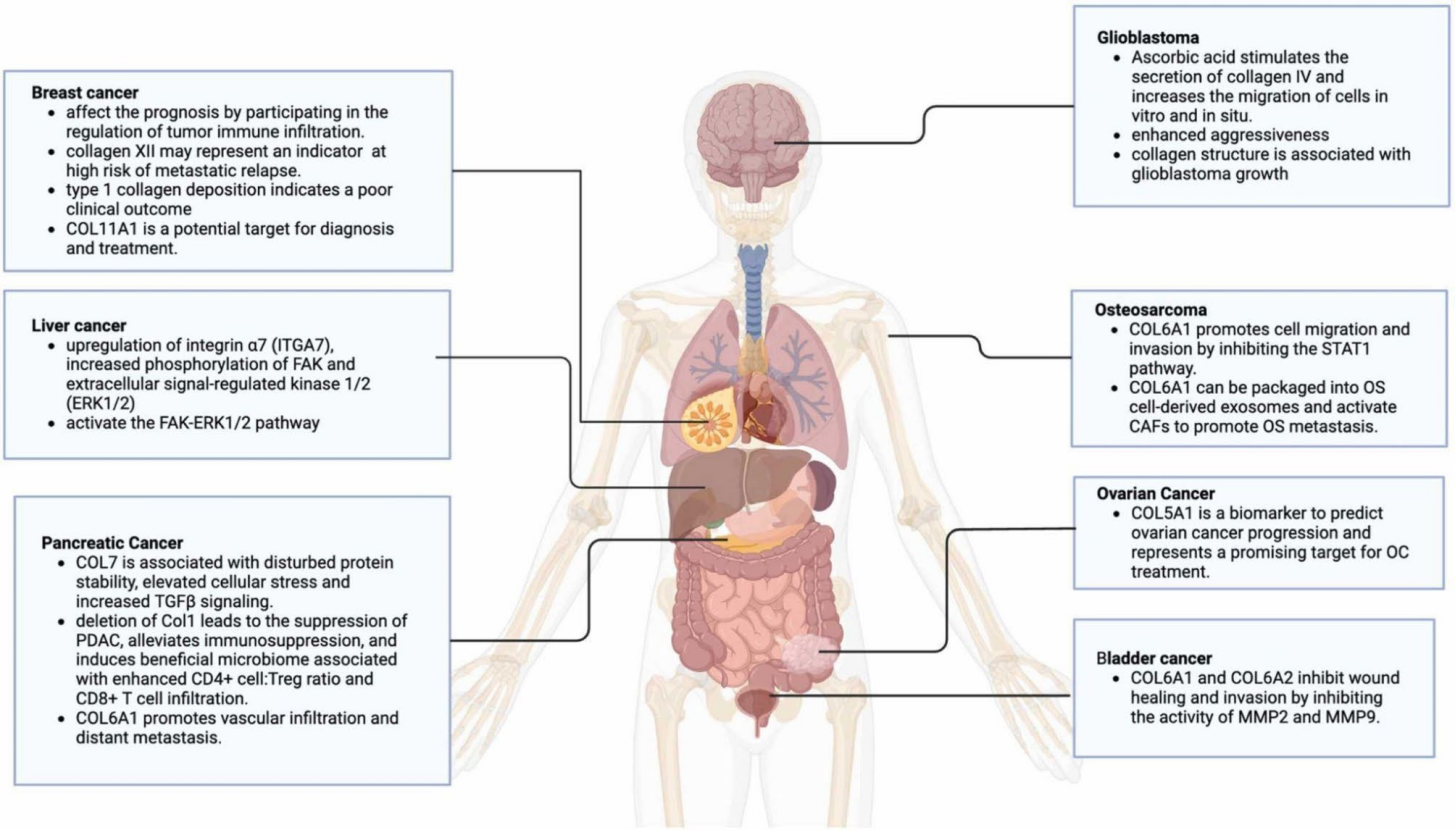
Collagen plays a critical role in the development and progression of various tumors by influencing tumor behavior through multiple signaling pathways and cytokines. Collagen expression is frequently upregulated in many cancers, and this elevated expression often serves as an independent risk factor for reduced overall survival across different cancer types. For example, type XI collagen (COL11A1) significantly impacts the prognosis of breast cancer patients by modulating the immune cell infiltration within tumors. Malignant tumors also exhibit greater stiffness compared to benign tumors, primarily due to the proliferation of connective tissue in the ECM, with COL I being the dominant structural component [5]. Figure adapted from Fig. 2 of Zhang et al. [5]

Collagens are fundamental components of the ECM, constituting approximately 90% of its composition in humans [94]. These proteins play a pivotal role in maintaining tissue integrity, providing mechanical support, and mediating cell signaling [95, 96]. Collagen signaling influences key functions, including growth, migration, and adhesion, which are critical for both normal tissue maintenance and cancer progression. Figure 2 illustrates various collagen signaling pathways involved in cancer progression.

The collagen family consists of approximately 28 distinct proteins [97], which undergo extensive modifications during growth, development, wound healing, and pathological processes, including cancer. The production of collagen involves the formation of disulfide bonds and other post-translational modifications. ECM molecules, such as fibronectin, play a crucial role in collagen placement and adherence within the ECM [58, 98]. Altered collagen levels and deposition, particularly of type I collagen (COL I), have been linked to developmental disorders and the onset of cancer (Table 2).

In healthy tissues, collagen organization is uniform, ensuring consistent ECM properties. However, in pathological conditions, collagen becomes heterogeneous, leading to altered ECM elasticity and the availability of growth factors [58, 97]. Tumor-Associated Collagen Signatures (TACS) have been identified as key indicators of pathological collagen remodeling and tumor progression. These signatures include TACS1: increased collagen deposition around the tumor, TACS2: spherical alignment of collagen layers surrounding the tumor, and TACS3: development of perpendicular collagen tracts extending away from the tumor [99]. These TACS patterns are strongly associated with cancer aggressiveness and poor prognosis.

Collagens also play a vital role in the structure of basement membranes, which are critical for separating tissue layers. Excessive collagen deposition can stiffen these membranes, impairing molecular exchange, while reduced collagen deposition thins the membranes, facilitating cancer cell migration, as demonstrated in vitro [58, 97].

**Table 2:** Roles of collagen subtypes in Cancer progression

Collagen subtype	Role in cancer	References
Type I (COL I)	- Most abundant collagen in ECM and solid tumors. - Supports tumor metabolism via stromal cell-mediated degradation into glutamine. - Aligned fibers promote tumor invasion; disorganized fibers act as barriers. - Increased levels correlate with larger tumor volumes and metastasis. - Targeting COL I (e.g., TEM8-neutralizing antibodies) disrupts cancer metabolism and reduces progression.	[72, 97, 99, 102–104]
Type II (COL II)	- Does not show prognostic value in cancer progression or treatment outcomes. - No significant correlation with tumor size or survival in pancreatic cancer.	[105]
Type III (COL III)	- Elevated levels correlate with larger tumor volumes and metastasis. - Decreased COL III levels, along with COL I reductions and increased COL IV, improve progression-free survival. - Found in metastatic tissues, contributing to tumor ECM stiffness.	[75, 97, 105, 119]
Type IV (COL IV)	- Major component of basement membranes; provides structural support. - Decreased levels in metastatic tissues but elevated in non-metastatic tissues. - Increases in COL IV are associated with improved treatment outcomes and smaller tumor sizes. - Loss of COL IV reduces ECM integrity, promoting migration and metastasis.	[97, 105, 119]
Type V (COL V)	- COL5A2 gene is upregulated in CRC epithelial cells, correlating with poor prognosis. - Promotes cancer cell growth via Wnt/β-catenin and PI3K/mTOR pathways. - Potential target for therapeutic interventions and biomarkers for prognosis.	[142]
General collagen	- Remodeling of collagen (e.g., via MMPs, LOXL-2) alters ECM stiffness, supporting tumor invasion and metastasis. - Hypoxia and inflammatory cytokines increase collagen deposition and crosslinking. - Platelet-collagen interactions promote angiogenesis and metastasis.	[119, 127, 129–133]

#### COL I in the tumor microenvironment

COL I, the most abundant collagen type, is highly prevalent in solid tumors, where it serves as a critical structural and metabolic resource [100]. Although tumor cells cannot directly utilize collagen due to the resistance of its triple-helical structure to proteolysis [101, 102], studies have shown that metabolic byproducts from collagen degradation, mediated by stromal cells, support cancer cell survival under nutrient-limited conditions. Stromal cells within the TME degrade collagen into glutamine, which cancer cells metabolize. Genetically modified mouse models have demonstrated that blocking collagen uptake-facilitated by the TEM8/ANTXR1 protein on stromal cells-using TEM8-neutralizing antibodies disrupts this survival mechanism, underscoring collagen’s role in tumor metabolism [73].

Beyond its metabolic role, COL I contributes significantly to the structural integrity of the tumor stroma [5, 99], influencing processes such as cell adhesion, migration, and proliferation [5]. The organization and alignment of COL I fibers can either facilitate or restricte tumor invasion and metastasis [103, 104]. Aligned collagen fibers act as migration tracks for tumor cells [102, 103], while disorganized networks can serve as physical barriers [102].

Additionally, COL I modulates signaling pathways through interactions with integrins and other cell surface receptors, thereby regulating cancer progression and stromal responses [103].

#### Profiling collagen subtypes in cancer

Profiling collagen subtypes provides valuable insights into cancer progression and treatment outcomes. A study analyzing residual tumor volume in relation to collagen subtypes using immunofluorescence after treatment initiation uncovered important correlations. For example, in pancreatic cancer, higher levels of COL I and COL III were associated with larger residual tumor volumes, while COL IV demonstrated an inverse relationship. COL II did not exhibit prognostic value, as it showed no correlation with treatment outcomes. Reductions in COL I and COL III, alongside increases in COL IV, were linked to smaller tumor volumes during disease progression. Moreover, after treatment initiation, inverse correlations were observed for COL I, COL II, and COL III, where higher percentages of these collagen subtypes were associated with shorter progression-free survival. In contrast, COL IV exhibited a direct correlation, suggesting that reductions in COL I, II, and III, combined with increased COL IV, could serve as predictive markers for prolonged progression-free survival [105].

This approach to collagen profiling offers the potential to optimize and personalize combination treatments based on tumor biopsies, improving the management of cancer progression and metastasis [105–109].

Inhibiting COL I synthesis has shown anti-tumor effects in animal models [84] and its fragments have potential as diagnostic markers for metastatic tumors with prognostic significance [84, 110, 111]. However, these effects are tumor type-dependent and may vary systemically. For example, combining Losartan- which improves tumor blood flow [112, 113] and reduces hypoxia [81, 114]-with the chemotherapy drug Doxil, an agent that blocks topoisomerase 2 necessary for cancer cells division [115], significantly reduced pancreatic tumor size in mice [116, 117].

Nevertheless, collagen’s role in cancer is complex, as it can have both pro- and anti-neoplastic effects depending on the type of tumor. For instance, while COL I inhibition has demonstrated therapeutic benefits, its deletion in pancreating cancer myofibroblasts was shown to accelerate tumor progression [83, 118 ].

#### Collagen remodeling in tumors

A study comparing collagen remodeling and collagenase activity in colorectal cancer (CRC) patients revealed that changes in collagen fiber arrangement are strongly associated with prognosis [119]. Tumor progression involves abnormal collagen remodeling, where collagen fibers in primary tumor matrices are curly and irregular, while in metastatic tissues display dense, linear, and directionally aligned fibers. CRC tissues show increased levels of COL I and COL III, which correlate with metastasis, whereas COL IV levels are higher in non-metastatic tissues but lower in metastatic tissues compared to normal tissues [119].

Elevated expression of MMPs, such as MMP-1 [120], MMP-2 [121], MMP-7 [122], and MMP-9 [123, 124], as well as lysyl oxidase-like (LOXL)-2 [125, 126] has been observed in the cancer stroma. These enzymes contribute to hyperactive collagen remodeling, tumor stage progression, and ECM degradation [119, 127]. Collagen fiber organization is also influenced by mechanical, biochemical, and genetic factors. Interactions between tumor cells and stromal cells, including CAFs and myofibroblasts, align collagen fibers, increasing ECM stiffness and supporting tumor invasion [122, 128].

Changes in collagen fiber arrangement are characterized by TACS, which reflect distinct patterns of collagen remodeling correlated with tumor progression. Growth factors like TGF-β and VEGF, along with inflammatory cytokines, play key roles in promoting collagen deposition and remodeling [128–131]. Hypoxic conditions within tumors activate hypoxia-inducible factors (HIFs), which drive increased collagen production and crosslinking. Additionally, the acidic tumor microenvironment enhances the activity of ECM-modifying enzymes [5, 132, 133].

Immune cells, particularly TAMs, release cytokines and proteases that alter collagen deposition, leading to ECM stiffening. Altered gene expression and epigenetic changes in both tumor and stromal cells significantly affect collagen synthesis and remodeling [2, 66, 134]. Hormonal signaling, such as the influence of estrogen and progesterone in hormone-sensitive cancersm further impacts collagen organization [135]. Paracrine signaling between tumor and stromal cells also plays a role in shaping collagen structure [136, 137]. MicroRNAs regulate the expression of ECM-related genes, driving changes in collagen metabolism [138–140].

Collectively, these factors shape the structure and dynamic of collagen fibers within the tumor ECM, ultimately supporting tumor progression, invasion, and metastasis.

#### Collagen and platelet activation

Gene Ontology (GO) and Kyoto Encyclopedia of Genes and Genomes (KEGG) analyses suggest that collagen promotes tumor development by activating platelets. Collagen or collagenases expressed on the surface of tumor cells bind to integrins on the platelet surfaces, triggering platelet activation. Once activated, platelets release growth factors that enhance tumor angiogenesis, facilitating tumor growth.

Beyond promoting angiogenesis, platelet binding to cancer cells protects these cells from shear-induced damage during circulation, thereby supporting successful cancer cell colonization. Abnormal collagen remodeling, along with related proteins and genes, is strongly linked to CRC tumorigenesis and metastasis, significantly influencing patient prognosis [119].

#### Therapeutic strategies targeting collagen

Collagen promotes CRC stemness and metastasis through the integrin/PI3K/Akt/Snail signaling pathway. Elevated COL I expression has been observed in CRC tissues from patients with high metastasis rates. CRC cells cultured on 2D collagen demonstrated significantly enhanced metastatic potential and tumorigenesis in both in vitro and in vivo models. Activation of the PI3K/Akt signaling pathway via integrin α2β1 increased the metastatic potential and stemness of CRC cells, with Snail serving as a downstream effector. Notably, blocking this pathway with E7820, an anti-cancer sulphonamide, successfully reversed COL I-induced distant metastasis in CRC [141].

The A3 domain of von Willebrand factor (vWF A3) acts as a collagen-binding domain, facilitating specific interactions with collagen. A synthetic CBD-SIRPαFc conjugate has been developed to block CD47, shielding cancer cells from phagocytic clearance while enhancing tumor-targeting capabilities via the collagen-binding domain. This conjugate has demonstrated improved anti-tumor efficacy by increasing MHC II + M1 macrophages [46]. Additionally, the vWF A3-SIRPαFc fusion protein exhibited superior tumor accumulation and retention compared to SIRPαFc alone. In an MC38 allograft model, the fusion protein showed enhanced tumor-suppressing effects, characterized by increased infiltration of MHC II + M1 macrophages and CD4 + T cells. These findings suggest that vWF A3 enhances the anti-tumor efficacy and immune-activating properties of SIRPαFc, reinforcing tumor collagen as a promising therapeutic target [127].

The COL5A2 gene is significantly upregulated in various CRC types, with its mRNA expression correlating with cancer stages, gender, recurrence, microsatellite instability, and KRAS mutation status. COL5A2 protein levels are elevated in cancer epithelial cells and correlate with age and T stage, whereas its stromal expression remains relatively unchanged between cancerous and normal tissues. Both gene and protein expressions of COL5A2 in epithelial cells are independent risk factors for poor CRC prognosis. Ectopic COL5A2 expression promotes colon cancer cell growth and enhances the Wnt/β-catenin and PI3K/mTOR signaling pathways through its interaction with discoidin domain receptor tyrosine kinase 1 (DDR1), suggesting its potential as a prognostic marker and therapeutic target in CRC [142].

Steen et al. [143] identified the cancer ECM as a relatively stable therapeutic target compared to the unstable nature of cancer cells. They proposed targeting the highly sulfated chondroitin matrix (CS-E) in cancer-associated stroma as a novel approach for drug delivery. Immunohistochemistry revealed CS-E-rich stroma in various solid tumors, highlighting its potential for matrix-based therapies.

Conversely, COL IV and metalloproteinase inhibitor-3 (TIMP-3) expression decreases as tumor stage advances. Additionally, cancer cell proliferation within the CRC ECM is higher compared to normal colon ECM, indicating that ECM disruption during tumor development creates a favorable microenvironment for cancer growth [144].

In summary, collagen in the ECM plays an integral role in tumor progression, metastasis, and resistance to therapy. By targeting collagen synthesis, remodeling, and associated pathways, cancer therapies can reduce ECM stiffness, impair metastasis, improve drug delivery, and enhance patient outcomes. The dual role of collagen, as a structural scaffold and a regulator of cell signaling, offers diverse opportunities for therapeutic intervention, making it a promising focus in cancer treatment research [82–86, 119, 143]. Table 3 summarizes the roles of collagen subtypes in cancer and associated treatment strategies.

**Table 3:** Overview of therapeutic strategies targeting collagen in Cancer treatment

Therapeutic strategy	Description	References
COL I inhibition	- Inhibiting COL I synthesis has shown anti-tumor effects in animal models. - Tumor type-specific outcomes.	[83, 110, 111]
Combination therapy (Losartan + Doxil)	- Losartan (improves blood flow) and Doxil (chemotherapy drug) reduce pancreatic tumor size in mice.	[116, 117]
Blocking integrin/PI3K/Akt/Snail pathway	- Blocking the pathway reduces COL I-induced metastasis in CRC, reversing distant metastasis.	[141]
vWF A3-SIRPαFc fusion protein	- A synthetic fusion protein improves tumor-targeting and enhances immune-activating properties. - It increases macrophage infiltration and tumor suppression.	[127]
Targeting COL5A2 in CRC	- COL5A2 upregulation in CRC correlates with poor prognosis. - COL5A2 promotes CRC cell growth and enhances cancer cell signaling pathways.	[142, 143]
Targeting ECM components (e.g., CS-E Matrix)	- Targeting CS-E-rich stroma can be a strategy for targeted drug delivery in various solid tumors.	[143]
COL I and metalloproteinase inhibitors	- Reducing COL I expression can improve the TME. - COL I and TIMP-3 expression changes are linked to CRC progression.	[144]

### Laminin

Laminin, a trimeric glycoprotein found in the basal lamina, is composed of α (α1 to α5), β (β1 to β3), and γ (γ1 to γ3) chains. It plays a crucial role in cell adhesion, migration, and differentiation by interacting with integrin receptors on cell surface [145, 146], self-polymerizing [147, 149], and conferring resistance towards apoptosis [148, 149]. Laminin also connects various ECM components and is essential for basement membrane assembly. In cancer, laminins, particularly laminin 332 (LN 332), are implicated in cancer stem cell self-renewal and drug resistance [150]. LN332 is associated with poorer survival outcomes in cancers like CRC [151] and pancreatic cancer [152] as it enhances cell adhesion, migration, and activates survival signaling pathways, such as mTOR, through interactions with integrins [56].

#### Laminin subunits and their role in Cancer

LAMA5, a subunit of laminin α5, is overexpressed in various human cancers, including pancreatic adenocarcinoma [153], CRC [154], non-small cell lung cancer (NSCLC) [155], and breast cancer [156]. Elevated LAMA5 expression has been observed in ovarian cancer tissues, where it correlates with International Federation of Gynaecology and Obstetrics (FIGO) staging and tumor grade. FIGO is a staging system for cervical cancer based on the size of cancer, location and spread, higher stages indicate more advanced disease and a less optimistic outlook. High LAMA5 expression is linked to poor overall survival, highlighting its prognostic significance [157].

LAMA5 plays a critical role in EMT, a key process in cancer progression that transforms epithelial cells into mesenchymal cells, facilitating cell invasion, metastasis and resistance to apoptosis. EMT is regulated by the Notch pathway, which promotes cell motility and invasiveness. Downregulation of LAMA5 reduced Notch protein expression, inhibiting cancer cell invasion and metastasis. This suggests that LAMA5 drives cancer cell progression by modulating EMT through the Notch pathway [157].

In KRAS-mutant hepatic metastases from CRC, LAMA5 is overexpressed in both tumor cells and the TME. LAMA5 and its receptor, BCAM, function at the interface between tumor cells and the TME, contributing to metastatic colonization. High vascular levels of LAMA5 enhances the adhesion of CRC cells to endothelial cells, promoting metastatic growth in the liver. Inhibition of the LAMA5/BCAM interaction disrupts this adhesion, reduces vascular colonization, and limits metastatic progression, underscoring the therapeutic potential of targeting this pathway [154].

#### Laminin in angiogenesis and tumor vascularity

LAMA5 expression is associated with increased vessel density and angiogenesis in hepatic metastases. Laminin 511 (LN511) (comprising LAMA5, LAMB1, and LAMC1) is localized in the vascular basement membrane and supports tumor vasculature. Deficiency in LAMA5 reduces vessel branching and efficiency, leading to hypoxia, which can accelerate tumor progression. While decreased LAMA5 may normalize blood vessels and potentially improve chemotherapy response through enhanced perfusion, reduced perfusion is also observed.

Downregulation of LAMA5 activates the Notch signaling pathway, which inhibits angiogenesis by suppressing Vascular Endothelial Growth Factor Receptor (VEGFR)-2 in stalk cells. Stalk cells, which maintain connectivity with parental vessels and form the trunk of new vessels during angiogenesis [158–160], exhibit a stalk-like phenotype in LAMA5-deficient endothelial cells, as indicated by elevated Hey2 expression, a key downstream mediator of Notch signalling [161].

#### Laminin 332 (LN332) and its clinical implications

LN 332, encoded by LAMA3, LAMB3, and LAMC2 genes, is integral to epithelial tissue homeostasis, contributing to cell adhesion, polarity, proliferation, and differentiation. It also supports tissue repair and regeneration by promoting cell migration and basement membrane assembly [147].

In cutaneous squamous cell carcinoma (SCC) cells, CAF-derived TGF-β stimulates LN332 production [145]. LN332 also contributes to immune evasion, as LAMC2 prevents T-cells infiltration into tumor tissues [162, 163]. Huang et al. [155] demonstrated that LAMC2 expression correlates with poor clinical outcomes in CRC, including overall survival, disease-free survival, and recurrence-free survival. LAMC2 expression is elevated in tumors compared to adjacent normal tissues and further increases in metastatic lesions. Overexpression of LAMC2 in CRC cells increases proliferation, migration, invasion, and S-phase cell proportions, as shown in Cell Counting Kit-8 (CCK-8) and transwell assays. Its correlation with Tumor, Node, Metastasis (TNM) staging suggests LAMC2 as a potential prognostic biomarker and therapeutic target in CRC.

#### Laminin Α1 (LAMA1) in tumor-stroma interactions

LAMA1, the α1 subunit of laminin, plays a pivotal role in tumor-stroma interactions. High LAMA1 expression increases colon tumor incidence, angiogenesis, and growth by recruiting carcinoma-associated fibroblasts, upregulating VEGFA expression via the integrin α2β1-CXCR4 complex, and binding of VEGFA to laminin 111 (LN 111), which promotes angiogenesis and tumor cell proliferation. A gene signature compromising LAMA1, ITGB1, ITGA2, CXCR4, and VEGFA is associated with poor prognosis in colon cancer [164].

In esophageal squamous cell carcinoma (ESCC), LAMA1 counteracts the tumor-suppressive effects of microRNA-202 (miR-202), which inhibits tumor growth and induces apoptosis by downregulating LAMA1, p-focal adhesion kinase (FAK), and p-Akt expression [165–167]. Laminin’s activation of the PI3K/Akt signaling pathway highlights its role in tumor development and resistance to anticancer therapies [151, 168].

#### Laminin as a therapeutic target

Targeting laminin in the ECM offers a promising therapeutic approach for cancer treatment. Laminin, particularly LN332 and LAMA5, plays critical roles in cancer cell invasion, metastasis, and resistance to apoptosis, contributing to tumor aggressiveness and drug resistance. Laminin-integrin interactions and pathways such as Notch and PI3K/Akt promote EMT, enhancing cell motility and survival. Laminin also drives angiogenesis, creating a favorable TME.

Disrupting laminin interactions can weaken tumor-stroma connections, reduce metastasis, and improve chemotherapy efficacy. Laminin’s role as a biomarker for poor prognosis further underscores its potential in targeted cancer therapies. Table 4 summarizes potential therapeutic strategies targeting laminin in cancer.

**Table 4:** Potential therapeutic strategies targeting laminin in cancer

Treatment strategy	Overview	References
Targeting laminin for metastasis inhibition	Laminin, particularly LN332 and LAMA5, promotes metastasis by facilitating cell adhesion, migration, and invasion. Inhibiting LAMA5/BCAM interactions reduces metastatic progression.	[154, 157]
Targeting Laminin in Angiogenesis	LAMA5 influences angiogenesis and vessel formation, contributing to tumor growth. Downregulation of LAMA5 can normalize blood vessels, potentially enhancing chemotherapy responses.	[158, 161]
Inhibition of Laminin in Tumor-Stroma Interactions	LAMA1 contributes to tumor growth and angiogenesis. Targeting the integrin α2β1-CXCR4 complex and VEGFA could disrupt tumor-stroma interactions, reducing tumor proliferation.	[164, 168]
Targeting Laminin for Immune Evasion	LAMC2, a component of LN332, inhibits T cell infiltration, facilitating immune evasion. Targeting LAMC2 could potentially improve immune responses in tumors.	[162–163, 155]
Inhibiting Laminin-Integrin Pathways for Drug Resistance	Laminin interactions with integrins activate survival signaling pathways, such as PI3K/Akt, promoting drug resistance. Disrupting these pathways can enhance chemotherapy sensitivity.	[151, 167, 168]
Targeting Laminin for Tumor Suppression	Laminin’s role in activating the PI3K/Akt pathway promotes tumor growth and resistance to therapies. Targeting laminin can inhibit these pathways, reducing tumor progression.	[167, 168]
Laminin as a Prognostic Marker	Laminin subunits, particularly LAMC2 and LAMA5, correlate with poor clinical outcomes in cancers like CRC, suggesting laminin’s potential as a prognostic biomarker.	[152, 155, 157]

### Fibronectin

Fibronectin (FN) is an ECM protein that regulates essential cellular processes, including cell adhesion, spreading, migration, proliferation, and apoptosis [61, 169]. FN typically exists as a dimer convalently linked by disulfide bonds, with each monomer compromising three types of repeating subunits: 12 type I (FN1), 2 type II (FN2) and 15–17 type III (FN3). FN1 and FN2 consist of β-pleated sheets stabilized by disulfide bonds, while FN3 adopts a seven-stranded β barrel structure that is susceptible to mechanical deformation. FN interacts with a range of binding partners, including integrins, collagen, heparin, fibrin, MMPs, and growth factors, enabling its versatile biological functions [170].

FN is assembled by cells into viscoelastic fibrils capable of binding up to 40 distinct growth factors and cytokines. These fibrils form a provisional ECM during embryonic development and share similarities with tumorigenesis [171], involving the p53 pathway [172, 173], as well as with wound healing. Tumors often exploit wound-healing mechanisms for growth [174]. FN fibril assembly, which is often upregulated in diseases such as cancer and fibrosis, facilitates mechanical signal transduction into biochemical cues [170].

The FN receptor, integrin α5β1 plays a central role in cell adhesion and migration. Dysregulation of integrin α5β1 is implicated in aggressive cancer phenotypes, promoting uncoordinated migration and metastasis [175]. FN binding to integrins α5β1 induces integrin clustering, focal adhesion formation, and intracellular signaling [176, 177]. These processes contribute to ECM remodeling, exposing additional binding sites for collagens and laminins, which further strengthen cell adhesion and facilitate migration [178]. This exacerbates disease progression by promoting tumor invasion and metastasis [61, 179].

#### FN isoforms and cancer progression

Isoforms of FN, particularly those containing ED-A and ED-B domains [177], are upregulated in the TME and are strongly associated with poor outcomes in cancers such as breast, colorectal, lung, ovarian, pancreatic, melanoma, and head and neck squamous cell carcinoma (HNSCC) (Table 5) [180]. These isoforms influence cell behavior by binding to cell surface receptors and inducing changes in acrtin filaments, thereby promoting cell movement [180, 183, 184]. Additionally, they enhance tumor cell adhesion, migration, invasiveness, ECM remodeling, and therapy resistance, establishing FN as a critical factor in cancer progression and metastasis [180–182].

**Table 5:** FN isoforms involved in different types of cancer

Cancer type	FN isoform	Prognostic implications	References
Breast cancer	ED-A, ED-B	- ED-A expressed in 50% of invasive ductal carcinomas, ED-B in 33%. - ED-B found in tumor vessels of 78% of invasive ductal breast carcinomas.	[183]
CRC	ED-A	- ED-A sustains CD133+/CD44 + subpopulation in CRC. - Correlates with lymph node metastasis, tumor differentiation, and clinical stage. - Positive staining for ED-A in tumor stroma and vessels.	[184–186]
Lung cancer	ED-A, ED-B	- ED-A induces EMT in lung cancer cells, promoting tumor progression. - No significant difference in ED-A expression between malignant and non-malignant tissues. - ED-A elevated in aged lungs and implicated in cancer. - Immunization against FN EDA decreased tumor burden and lung metastases in MMTV-PyMT model. - ED-B broadly expressed in lung cancer stroma.	[183, 187, 188]
Ovarian cancer	ED-A, ED-B	- Positive staining for ED-A in tumor stroma and vessels. - ED-B broadly expressed in ovarian cancer stroma.	[186–188]
Pancreatic cancer	ED-A, ED-B	- Positive staining for ED-A in tumor stroma and vessels. - ED-B found in the stroma of pancreatic cancer.	[187, 188]
HNSCC	ED-B	- ED-B broadly expressed in stroma of HNSCC.	[188]
Melanoma	ED-A	- Strong staining of ED-A in primary melanoma lesions at the basal lamina of the epidermal-dermal interface.	[183]

Integrin-mediated signaling is regulated by specific FN isoforms, collectively referred to as cellular FN. Unlike circulating plasma FN, cellular FN isoforms are generated through alternative splicing of the FN1 gene. These isoforms differ in solubility, receptor binding capacity, and spatiotemporal expression, making them highly adaptable to the dynamic requirements of tumor progression [180] (Table 6).

**Table 6:** Comparison of cellular FN and plasma FN

Feature	Cellular FN	Plasma FN
Structure	Longer, more flexible, contains additional ED-A and ED-B domains due to alternative splicing [189–191]	More compact and soluble, lacks ED-A and ED-B domains [192]
Function	Involved in cell adhesion, migration, and ECM remodeling [193]	Primarily involved in blood clotting, wound healing, and tissue repair [193]
Regulation and expression	Expression regulated by growth factors and environmental cues; upregulated during wound healing and fibrosis [193–195]	Continuously produced by the liver [196]; expression increases during inflammation or injury [197]

FN1 has been extensively studied for its involvement in tumor initiation [88], progression [198], metastasis [199], and response to chemotherapy [200, 201]. Knockdown of FN1 inhibits tumor growth [202, 203], while its upregulation promotes cell migration, invasion, cancer viability, adhesion [203, 204], and survival [205, 206] through the FAK signaling pathway [207–209].

Overexpression of FN1 increases the expression of anti-apoptotic proteins like Bcl-2 [202] and pro-metastatic factors such as MMP-2, MMP-9, N-cadherin [202]. At the same time, it reduces pro-apoptotic markers like Bax [205] and caspase-3 [210]. Zhou, Shua, and Huang [211] demonstrated that inhibiting FAK significantly suppresses Bcl-2 expression while increasing Bax levels and caspase-3 activity, regardless of FN1 upregulation. Reduced FAK and phosphorylated FAK (p-FAK) levels induce apoptosis by elevating Bax, enhancing caspase-3 activity, and supressing Bcl-2 expression. FAK activation is also essential for the upregulation of N-cadherin, MMP-2, and MMP-9, with reduced FAK expression correlating with lower levels of these molecules.

FN1 also interacts with integrin β1, specifically the α5β1 integrin heterodimer [203], a mediator of FN signaling. FN1 binding to integrin β1 facilitates activation of the Wnt/β-catenin signaling pathway. This process involves the phosphorylation and inactivation of Gsk3β, leading to the accumulation of active β-catenin, a transcriptional regulator. Silencing FN1 disrupts its interaction with integrin β1, significantly inhibiting Wnt/β-catenin signaling [204, 212]. Notably, the Wnt/β-catenin pathway is linked to increased sensitivity to chemotherapeutic agents [205, 213] but also contributes to chemotherapy resistance [214–216].

#### FN and resistance

Pretreatment of culture dishes with the general form of FN significantly promotes cancer cell proliferation, sustained tumor growth, and drug resistance both in vitro and in vivo. Mechanistically, FN binds to integrin αvβ1, activating the FAK/CDC42 signaling pathway. This supresses the phosphorylation of YAP-1, leading to its activation and nuclear localization. Nuclear YAP-1 upregulates the tumor-associated transcription factors like SOX2, driving cancer cell proliferation and drug resistance. Blocking the integrin αvβ1 signaling pathway significantly inhibits tumor growth and reduces chemoresistance [217, 218].

Patients experiencing failure of post-operative radiation therapy exhibit higher FN1 expression levels, identified as a marker of radiation resistance gene, associated with the ability of cells to resist radiation damage. FN1 overexpression correlates with poorer prognosis and is localized to the focal adhesion pathway. Elevated FN1 expression suggests that tumors adapt to radiation-induced changes, developing radioresistance. This makes FN1 a potential driver of focal adhesion pathway-associated radioresistance [219, 220].

Conversely, overexpression of PLA2R1, a regulator of cell proliferation, differentiation, and apoptosis, inhibits cancer progression. PLA2R1 competes with FN1 for binding to integrin β1, thereby disrupting downstream FAK signaling. This disruption inhibits malignant cell proliferation, highlighting PLA2R1 as a therapeutic target to counteract FN1-mediated cancer progression [208].

#### Therapeutic potential of FN targeting

Targeting FN and its signaling pathways offers a promising strategy for cancer therapy. FN-mediated interactions with integrins, particularly α5β1 and αvβ1, activate key pathways such as FAK, CDC42, and Wnt/β-catenin, driving tumor invasion, survival, and drug resistance. Inhibition of these pathways disrupts tumor-stroma interactions, reduces metastasis and enhances chemotherapy response.

FN isoforms containing ED-A and ED-B domains exacerbate disease progression by remodeling the ECM, facilitating metastatic spread, and inducing therapy resistance. Blocking these isoforms or their receptors may impair tumor growth and improve treatment outcomes.

In conclusion, FN’s pivotal role in cancer biology makes it an attractive therapeutic target. By disrupting FN’s interactions with integrins and downstream signaling pathways, it may be possible to reduce tumor growth, inhibit metastasis, and improve chemotherapy sensitivity. Table 7 provides an overview of FN isoforms, their roles in cancer progression and prognosis, and their therapeutic implications.

**Table 7:** The role of FN in Cancer progression and therapeutic targeting

Treatment strategy	Overview of potential approaches	References
Targeting FN isoforms (ED-A, ED-B)	Blocking FN isoforms containing ED-A and ED-B domains could disrupt tumor growth, inhibit metastatic spread, and reduce therapy resistance by affecting ECM remodeling.	[177, 180]
Targeting FN-integrin interactions	Inhibiting FN interactions with integrins, especially α5β1 and αvβ1, may disrupt signaling pathways (FAK, CDC42, Wnt/β-catenin) and reduce tumor invasion, survival, and resistance.	[176, 178, 212, 217]
Inhibiting FAK/integrin signaling pathways	Disrupting the FAK/CDC42 signaling pathway through FN inhibition could impair tumor growth, reduce metastasis, and sensitize cancer cells to chemotherapy	[217, 218, 207,–209]
FN1 targeting to overcome chemoresistance	Blocking FN1’s interaction with integrin β1 can suppress the Wnt/β-catenin signaling pathway, reducing chemoresistance and enhancing the sensitivity of cancer cells to chemotherapy.	[205, 212–216]
Targeting FN-mediated angiogenesis and tumor vascularity	Targeting FN-mediated angiogenesis, through inhibition of FN or its isoforms, may reduce tumor vasculature, hinder metastasis, and enhance the delivery of chemotherapeutics.	[154, 161, 188]
Inhibition of PLA2R1 in FN1-driven cancer progression	Targeting PLA2R1, which competes with FN1 for binding to integrin β1, may inhibit FN1-mediated cancer progression by disrupting the FAK signaling pathway.	[208]
Targeting FN for radiation resistance	FN1 upregulation is associated with radioresistance in tumors. Targeting FN1 may overcome radiation resistance, improving the efficacy of radiotherapy.	[219, 220]

### Periostin

Periostin (POSTN) is a key mediator of cellular and extracellular communication, influencing ECM organization and connective tissue dynamics. It plays critical roles in embryonic development, tissue repair, and the development of collagen-rich connective tissues, such as bone and teeth. Aberrant POSTN expression is frequently observed in solid epithelial tumors, where it drives tumor progression by interacting with cell-surface integrin receptors. This interaction impacts cancer hallmarks, including proliferation, invasion and metastasis and cell survival [206]. Elevated POSTN levels also affects the size and quantity of metastatic lesions, underscoring its role in modifying the TME [221].

#### Role of POSTN in cell migration and ECM dynamics

POSTN significantly influences cell migration by interacting with integrins such as αvβ3, αvβ5, and α6β4, activating the Akt/protein kinase B (PKB) and FAK signaling pathways. It regulates COL I fibrillogenesis [222], influencing the biomechanical properties of connective tissues within the ECM [223]. POSTN expression is regulated by inflammatory cytokines, including transforming growth factor-β (TGFβ), interleukin (IL)-4, and IL-13, which induce the expression of POSTN splice variants and related ECM proteins, including FN and tenascin-C. The secreted splice variants of POSTN interacts with integrin αvβ3 on fibroblastic cells, promoting migration, through Akt and FAK phosphorylation, stimulating COL I production, and aiding tissue repair [224].

#### POSTN and the TME

Studies reveal that POSTN-integrin-NF-κB signaling enhances tumor growth by promoting M2 macrophages and CAFs. Elevated POSTN expression in invasive ovarian cancer, co-localized with integrins β3 and β5, activates ERK and NF-κB pathways. POSTN’s autocrine effects stimulate cytokine secretion, attracting and polarizing THP-1 monocytes into M2 macrophages in vitro. Furthermore, tumors derived from POSTN-overexpressing SKOV3 cells exhibit increased TAMs and CAF-like cells, with higher CAF abundance correlating with advanced disease stages and poorer survival in metastatic tumors overexpressing POSTN [252].

#### POSTN in CAFs

Research by Yue at al. [225] demonstrated that POSTN derived from CAFs interacts with integrin receptors on ovarian cancer cells, activating PI3K/Akt pathway and inducing epithelial-mesenchymal transition (EMT). This promotes cancer cell migration and invasion. In gastric cancer, POSTN-expressing CAFs facilitate tumor invasion and metastasis by degrading the ECM and attracting M2-like macrophages, thus creating a metastatic niche within the TME [226].

In head adn neck squamous cell carcinoma (HNSCC), POSTN-expressing CAFs maintain the cancer stem cell phenotype by activating the protein tyrosine kinase 7 (PTK7)-Wnt/β-catenin signaling pathway, which is critical for sustaining stemness [227]. Similarly, in CRC, POSTN sustains the cancer stem cell niche, supporting stemness and metastatic colonization [228].

In melanoma, POSTN-expressing CAFs contribute to drug resistance. BRAF inhibitors activate B-linked proteins in CAFs, leading to increased POSTN secretion [222]. This upregulation enhances ECM stiffness, supporting tumor cell survival [64]. Mechanistically, POSTN activates the PI3K/Akt pathway, which reactivates the ERK pathway inhibited by BRAF inhibitors, promoting drug resistance [222, 229]. Additionally, POSTN recruits and activates CAFs, further contributing to resistance by secreting growth factors and remodeling the ECM [230, 231].

#### POSTN AND IL-6 feedback loop

The POSTN-IL-6 loop plays a pivotal role in regulating interactions between tumor cells and CAFs during colorectal tumorigenesis [232]. Interleukins (ILs), chemical signals that mediate communication between white blood cells and other cells and tissues, are central to this process [233]. Among them, IL-6 fosters a microenvironment conducive to cancer growth while also influencing the tumor-directed immune response [234]. IL-6 exhibits pro-inflammatory properties, stimulates tumor growth and angiogenesis, adn recruits immune cells. Paradoxically, it also contributes to an immunosuppressive environment that shields cancer cells from immune attack [235].

These effects are mediated through several signaling pathways, with signal transducer and activator of transcription 3 (STAT3) playing a key role [236]. Ma et al. [232] demonstrated that inhibiting STAT3 significantly reduces POSTN mRNA and protein levels, highlighting that IL-6 and STAT3 signaling regulate POSTN expression.

In turn, POSTN promotes the nuclear localization of yes-associated protein (YAP) and transcriptional coactivator with PDZ-binding motif (TAZ), which enhances IL-6 expression in tumor cells. Tumor-derived IL-6 activates STAT3 in CAFs, which boost POSTN secretion, thereby establishing a feedback loop. This loop drives IL-6 production, promotes tumor cell proliferation, and supports YAP/TAZ signaling activation in tumor cells [237, 238].

#### POSTN and immune invasion

POSTN contributes to immune evasion in the TME by binding to receptors such as integrins αvβ3, αvβ5, and α6β4, inducing intracellular signaling via the FAK, Akt, JNK and NF-κB pathways. Notably, POSTN-induced expression of programmed death-1 (PD-1) is abolished when cells are pre-incubated with antibodies targeting integrins αvβ3 and αvβ5, or with NF-κB inhibitors. This highlights the central role of NF-κB in regulating PD-1 expression as well as the production of IL-6 and interferon-gamma (IFN-γ) [239].

POSTN promotes PD-1 expression on tumor-associated macrophages (TAMs) via integrin-ILK-NF-κB signaling, which leads to increased production of IL-6 and IFN-γ. These cytokines, in turn, induce PD-L1 expression on colorectal tumor cells, contributing to an immunosuppressive TME [239].

Importantly, the combined inhibition of POSTN and PD-1 demonstrates significant suppression of colorectal cancer (CRC) progression compared to inhibiting either POSTN or PD-1 alone. This was demonstrated in experiments utilizing two plasmid vectors: one expressing periostin-specific small interfering RNA (siRNA) (pSi-periostin) and the other expressing PD-1-specific siRNA (pSi-PD-1). Together, these vectors effectively reduced the expression of both POSTN and PD-1, showcasing a synergistic therapeutic effect [239].

#### POSTN and ECM remodeling

POSTN promotes extracellular matrix (ECM) remodeling by binding to ECM molecules such as collagen, tenascin C, and fibronectin. This remodeling alters ECM stiffness and integrity, supporting tumor cell migration and invasion. In cervical squamous cell carcinoma (CSCC), cancer-associated fibroblasts (CAFs) with high POSTN expression (POSTN + CAFs) in primary tumors and metastatic lymph nodes correlate with lymph node metastasis and poor survival.

Mechanistically, POSTN + CAFs impair lymphatic endothelial barriers via the integrin-FAK/Src-VE-cadherin signaling pathway, facilitating tumor cell metastasis. Notably, inhibition of the FAK/Src pathway alleviates this barrier dysfunction, restoring endothelial integrity and reducing metastatic potential.

In contrast, CAFs with low or no POSTN expression (POSTN-CAFs) do not impair endothelial barrier function, which may explain the absence of lymph node metastasis in some cases. These findings identify a specific subset of POSTN + CAFs that actively promotes lymph node metastasis, highlighting the potential of targeting POSTN-expressing CAFs or their associated signaling pathways as a therapeutic strategy to prevent or mitigate metastasis in CSCC [240].

#### POSTN and chemoresistance

POSTN significantly contributes to chemoresistance by activating key signaling pathways such as PI3K/Akt and ERK. In epithelial ovarian carcinoma, POSTN-induced Akt phosphorylation drives resistance to paclitaxel, while in gemcitabine-treated cancers, POSTN enhances resistance by increasing Akt and ERK phosphorylation [241, 242]. Furthermore, POSTN promotes cancer cell proliferation and migration, effects inhibited by anti-POSTN peptides. For instance, in breast cancer, these peptides reverse doxorubicin resistance by modulating Akt phosphorylation and reducing survivin expression, a key apoptosis inhibitor [243]. Elevated POSTN levels in drug-resistant cancer cell lines compared to parental cells underscore its critical role in mediating chemotherapy resistance.

POSTN also influences epithelial-mesenchymal transition (EMT) via ERK activation, enhancing migratory and invasive capabilities in vitro. These effects can be suppressed through POSTN knockdown [244]. By inducing EMT, POSTN facilitates the transformation of cancer cells into more aggressive phenotypes, further contributing to resistance [225].

Moreover, POSTN deficiency reduces infiltration of PD-1 + tumor-associated macrophages (TAMs) in the tumor microenvironment (TME) and enhances TAM-mediated phagocytosis of tumor cells. A combined blockade of POSTN and PD-1 demonstrates significant tumor growth suppression, reducing PD-1 + TAM infiltration while increasing CD8 + T-cell presence. This synergistic approach highlights the potential of targeting POSTN alongside immune checkpoint inhibitors like PD-1 to overcome adaptive resistance [239].

#### Therapeutic potential of POSTN targeting

The PI3K/Akt pathway, which regulates cancer hallmarks such as cell survival, metastasis, and metabolism, is activated by PTEN, Akt, and mTOR [245]. Akt downregulates transcription factors FOXO2 and FOXO3a, which typically mediate survivin silencing via PTEN. Through the PI3K/Akt pathway, NF-ĸB upregulates survivin expression, while insulin-like growth factor-1 (IGF-1) stimulates survivin levels via PI3K/Akt/mTOR or by inactivating TGF-β, a negative regulator. Akt also supresses p53, further promoting survivin upregulation [246].

POSTN promotes to anticancer drug resistance by activating Stat3 and Akt and upregulating survivin expression [247]. In cisplatin-resistant lung cancer cells, elevated POSTN expression highlights its role in drug resistance. Survivin deleption significantly reduces POSTN’s protective effect against apoptosis, indicating that POSTN’s anti-apoptotic role involve caspase-3 inhibition [248].

High POSTN expression is closely linked to increased cell survival and proliferation, primarily mediated by integrins αvβ1, αvβ3, and αvβ5 [249]. POSTN facilitates ECM-cell interactions by binding to ECM molecules like collagens, tenascin C, and FN. These interactions regulate signaling cascades such as Notch 1 and β-catenin, which are critical for cell differentiation and tissue specification [61].

Recent studies suggest that POSTN serves as a prognostic marker in various cancers, including pancreatic [250, 251], ovarian [252, 253], and esophageal cancers [254]. Its multifaceted roles in ECM remodeling, signaling pathway activation, and TME modulation underscore its potential as both a therapeutic target and a prognostic indicator in cancer management.

In summary, POSTN plays a critical role in cancer progression, by influencing ECM dynamics, cell signaling, and the TME. Through its interactions with integrin receptors and activation of signaling pathways such as PI3K/Akt and NF-κB, POSTN promotes tumor cell proliferation, migration, and survival. It also contributes to chemotherapy resistance and immune evasion. Targeting POSTN offers a promising strategy to disrupt these processes, enhancing therapeutic efficacy, and overcome resistance in metastatic and aggressive cancers. Table 8 provides an overview of potential treatment strategies targeting POSTN in cancer therapy.

**Table 8:** Potential treatment strategies targeting POSTN in Cancer therapy

Treatment strategy	Overview	References	
Targeting POSTN Expression	Inhibition of POSTN expression or its interactions with integrins can disrupt tumor progression by reducing cell migration, invasion, and metastasis. This can potentially improve chemotherapy outcomes and reduce tumor aggression.	[113, 225, 239]	
Inhibition of POSTN-Integrin Interaction	Blocking POSTN-integrin interactions can inhibit key pathways like PI3K/Akt and FAK, reducing tumor growth, metastasis, and chemoresistance. This approach can target both primary and metastatic cancer cells.	[222, 225, 239]	
Combination with PD-1 Inhibition	Combining POSTN inhibition with PD-1 checkpoint inhibitors can significantly reduce tumor progression. This strategy targets immune evasion by reducing PD-1 expression on tumor-associated macrophages and enhancing T cell response.	[239]	
Inhibition of ECM Remodeling	Targeting POSTN’s role in ECM remodeling, by inhibiting its interactions with ECM proteins like collagen and fibronectin, can impair metastasis and reduce tumor cell migration and invasion.	[240]	
Targeting the POSTN-IL-6 Feedback Loop	Disrupting the POSTN-IL-6 feedback loop can reduce cancer cell proliferation and survival. Inhibition of IL-6 or its downstream pathways, including STAT3, could inhibit POSTN secretion and reduce tumor aggressiveness.	[232, 235–238]	
Chemoresistance Targeting	Inhibiting POSTN’s effect on pathways like Akt/ERK, which mediate drug resistance (e.g., paclitaxel, gemcitabine, doxorubicin), can restore the effectiveness of chemotherapy and reverse resistance in drug-resistant tumors.	[239–244]	
Targeting CAF-POSTN Signaling	Inhibiting POSTN derived from CAFs (cancer-associated fibroblasts) can suppress tumor growth by reducing ECM stiffness, inhibiting EMT, and restoring normal endothelial function to reduce metastasis.	[225–227]	
Targeting Multiple Pathways	Using multi-targeted therapies to block POSTN-induced activation of signaling pathways (e.g., PI3K/Akt, NF-κB, ERK) can enhance therapeutic efficacy and potentially overcome resistance in aggressive cancers.	[245–249]	

### Hyaluronic acid

Hyaluronic acid (HA), or hyaluronan, is a glycosaminoglycan composed of repeating disaccharide units of N-acetylglucosamine and D-glucuronic acid [255]. HA plays a pivotal role in cellular processes, including proliferation, migration, and invasion, through interactions with specific receptors and hyaladherins, such as CD44 and the receptor for hyaluronic acid-mediated motility (RHAMM) [256, 257].

In cancer, the reactive stroma is often characterized by an increase in CAFs and myofibroblasts, which produce various growth factors and chemokines that enhance the synthesis of HA and proteoglycans like versican. Interactions between versican, HA, and CD44 promote the expansion of the pericellular matrix, increasing its viscoelasticity and creating a supportive environment for cancer cell proliferation and migration [258].

#### HA-CD44 interacton in cancer progression

Witschen et al. [256] demonstrated that breast cancer cells synthesize and fragment HA while expressing CD44 on their surfaces, suggesting that HA-CD44 interactions contribute to cancer-associated inflammation and early tumor formation. Elevated expression of HA synthase 2 (HAS2) significantly correlates with an inflammatory gene signature, indicating that high levels of HA within tumors may serve as a poor prognostic marker [259, 260].

The interaction between HA and CD44 leads to the formation of HA/CD44 complexes on the cell membrane, activating intracellular signaling pathways that facilitate cell adhesion to the ECM and neighboring cells. This HA/CD44 axis plays a critical role in carcinogenesis by modulating pathways linked to cancer progression [261].

#### HA in gliomas and chemoresistance

In gliomas, upregulation of HAS2 and subsequent HA secretion are associated with increased cell proliferation, invasion, and chemoresistance via c-myc pathway. Targeting HAS2 has been shown to enhance glioma cell sensitivity to chemotherapeutic agents.

During tumor progression, significant changes in the ECM composition occur as cancer cells transition from pre-invasive to invasive phenotypes. These ECM alterations promote angiogenesis and inflammation, creating a mitogenic environment conducive to cancer progression. The HA-rich matrix enhances metastatic potential by improving cell motility and modifying biomechanical properties of tumor tissue, supporting cellular separation during mitosis, and promoting hydration and mobility [262].

#### HA and sulphated HA (sHA) in cancers

Research indicates that sulfated HA (sHA) is a potent inhibitor of prostate cancer. sHA reduces the proliferation, motility, and invasion in prostate cancer cell lines (e.g. LNCaP, LNCaP-AI, DU145, and LAPC-4) by inducing caspase-8-dependent apoptosis, downregulating anti-apoptotic proteins like Bcl-2, and inhibiting Akt signaling. sHA also affects androgen receptor phosphorylation, NF-κB activation, and VEGF expression. These effects are attributed to sHA blocking interactions between HA and its receptors (e.g. CD44 and RHAMM) and downregulating their expression [263].

In animal models, sHA significantly inhibits the growth of LNCaP-AI prostate tumors without causing significant weight loss or serum-organ toxicity. It also decreases tumor angiogenesis and enhances apoptosis, demonstrating its potential as a safe and effective cancer therapy.

### HA-CD44 and chemoresistance

In breast cancer, meta-analyses have linked elevated HA levels with poor overall survival and reduced disease-free, recurrence-free, and progression-free survival rates. High HA levels in stroma and plasma correlate with poor prognosis, including lymph node metastasis and higher tumor grade [264]. Similarly, coexpression of ex-synthase 3 (HAS3) in renal carcinoma is associated with metastasis and shorter survival [265].

HA-CD44 interactions regulate various cellular processes such as survival, growth, invasion, and metastasis. These interactions activate key signaling pathways, including RhoGTPases and the PI3K/AKT pathway, which play roles in chemoresistance [266].

Targeting HA in the ECM represents a promising approach due to its pivotal role in tumor progression, metastasis, and drug resistance. Through interactions with receptors like CD44 and RHAMM, HA drives processes such as proliferation, migration, and invasion. In cancers with reactive stroma, CAFs and myofibroblasts increase HA production, contributing to ECM remodeling, enhancing the TME viscoelasticity, and supporting cancer cell motility. Elevated HA levels promote inflammatory responses, cell adhesion, and signaling pathways that drive tumor growth and metastasis. Additionally, HA contributes to chemoresistance by activating pathways such as PI3K/AKT and RhoGTPases, which enhance tumor cell survival and drug resistance. Targeting HA synthesis or signaling could disrupt these processes, reduce tumor progression, and improve therapeutic efficacy, particularly in metastatic and chemoresistant cancers.

### Biological barriers for drug delivery and the role of peptides in overcoming them

Delivering drugs to cancer cells requires overcoming several biological barriers that impede the efficient transport of therapeutic agents. The first barrier is blood circulation, where particles interact with plasma proteins to form a protein corona, which alters their biodistribution and clearance. Upon reaching the tumor site, the dense and complex ECM presents another significant obstacle, limiting drug diffusion to cancer cells. After traversing the ECM, particles must cross the cell membrane, a lipid bilayer that is particularly challenging for hydrophilic or large molecules. Once inside the cell, therapeutic agents often become trapped and degraded within endosomes, making effective endosomal escape mechanisms essential for releasing the drugs into the cytoplasm. Furthermore, many therapies require precise targeting to specific organelles, such as the nucleus or mitochondria, for therapeutic efficacy - an additional challenge in drug delivery [267].

Peptides have emerged as versatile tools to overcome these barriers due to their biocompatibility, specificity, and functional adaptability. To address protein corona formation in blood circulation, peptide coatings can create a “stealth” layer that minimizes nonspecific protein adsorption and enhances circulation time [268]. For ECM penetration, tumor-penetrating peptides (TPPs), such as iRGD, bind to ECM components and trigger trans-tissue transport, while enzyme-activatable peptides cleaved by MMPs facilitate ECM degradation and localized drug release [269]. To cross the cell membrane, cell-penetrating peptides (CPPs), such as TAT and penetratin, enable receptor-independent translocation of t therapeutic agents. Endosomal entrapment can be mitigated with pH-responsive or fusogenic peptides that disrupt endosomal membranes in acidic environments or peptides that exploit the proton sponge effect, inducing osmotic swelling to release encapsulated drugs [270]. Additionally, organelle-specific targeting can be enhanced with specialized peptides: nuclear localization signals (NLS) direct therapeutic agents to the nucleus, while mitochondria-targeting peptides (MTPs), such as triphenylphosphonium-conjugated peptides, deliver drugs directly to the mitochondria for improved efficacy [271].

### Leveraging intelligent nanoparticle design to overcome ECM barriers

The ECM presents a major challenge in drug delivery due to its dense and complex structure, which limits nanoparticle penetration and drug diffusion. However, advances in intelligent nanoparticle design have enabled researchers to overcome these barriers through functionalization, stimuli-responsiveness, self-activating mechanisms, and multi-functional systems.

Functionalization strategies involve decorating nanoparticles with ECM-targeting ligands, such as HA or collagen-binding peptides, allowing for selective accumulation within tumor ECM regions. Additionally, protease-sensitive linkers, cleaved by MMPs, facilitate localized ECM degradation and trigger drug release at the tumor site [268, 269].

Stimuli-responsive nanoparticles further enhance drug delivery by exploiting the unique characteristics of the TME. For instance, pH-responsive nanoparticles utilize the acidic microenvironment of tumors to enable localized drug release. Similarly, thermo-responsive systems, such as β-cyclodextrin-based hydrogels, undergo structural changes under hyperthermic conditions to release therapeutic payloads effectively [270]. Self-activating nanoparticles, which respond to endogenous stimuli such as redox gradients or enzymatic activity, offer autonomous and precise drug release capabilities.

Multifunctional nanoparticles combine multiple strategies to overcome ECM barriers and optimize therapeutic outcomes These designs can simultaneously remodel ECM barriers and deliver drugs, reducing off-target effects and enhancing therapeutic efficacy. Some systems even incorporate immunomodulatory functions, which improve tumor accessibility while activating immune responses to support therapy [267].

## Conclusion

**Fig. 3:**
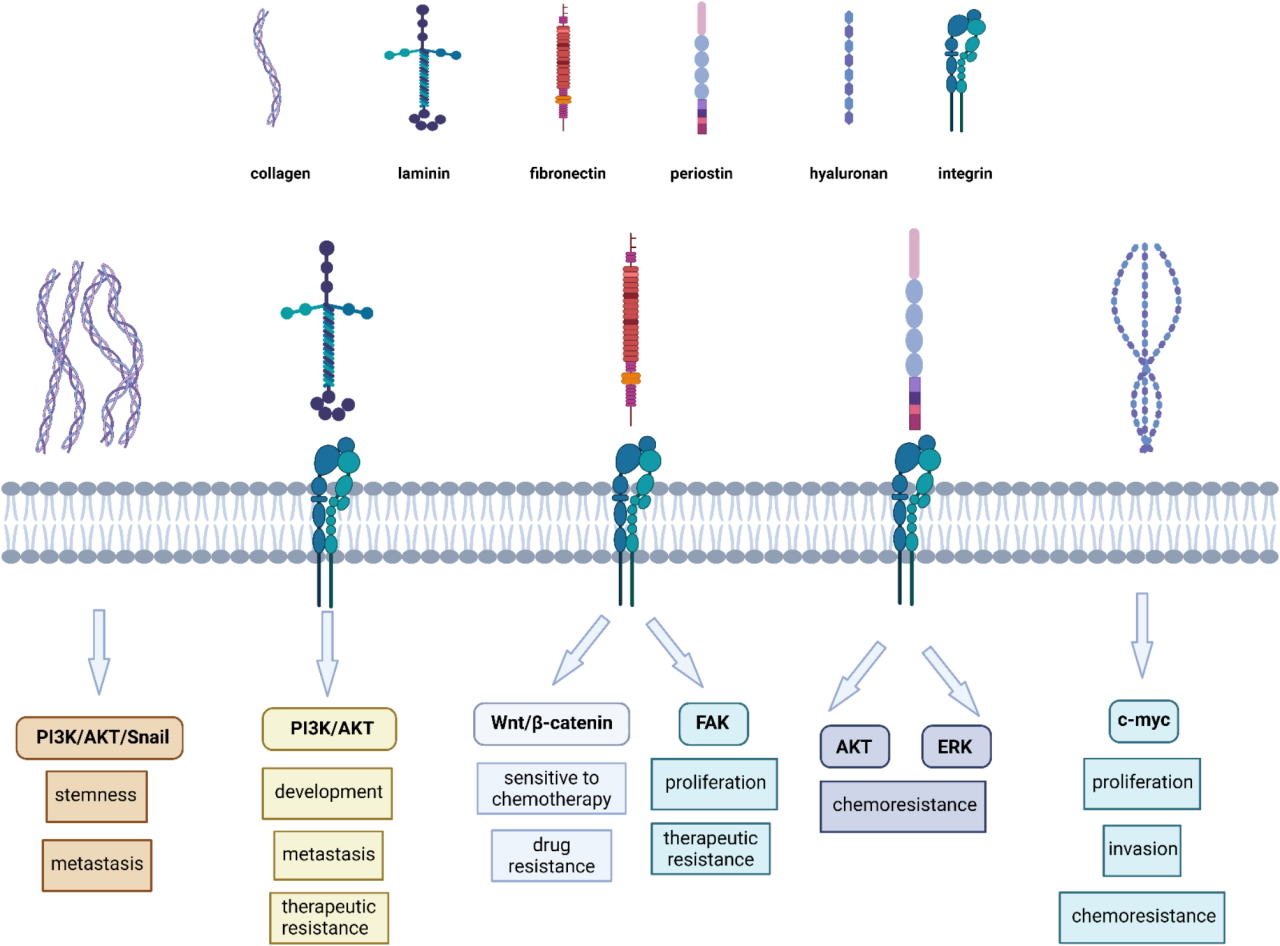
An overview of key signaling pathways involved in cellular processes highlights their highly regulated interactions, which coordinate essential cellular functions. In cancer, dysregulation or aberrant activation of these pathways is frequently observed, contributing to metastasis, resistance, and other complications. Understanding these pathways offers valuable insights into novel therapeutic strategies that target specific cellular processes, offering promising treatment options for diseases characterized by uncontrolled cell growth and progression. (Created with BioRender.com)

In conclusion, the ECM plays a central role in shaping the TME, influencing tumor progression, metastasis, and therapeutic resistance. Its complex biochemical, structural, and mechanical properties not only present significant barriers to effective cancer treatment but also act as key mediators of tumor-stroma interactions, angiogenesis, and immune evasion. Figure 3 highlights the signaling pathways involved in various cellular processes.

Recent advancements in ECM-targeted therapies and intelligent nanoparticle designs offer promising strategies to overcome these challenges. Targeting ECM components such as collagen, laminin, FN, and HA can disrupt tumor progression, reduce metastasis, and enhance drug delivery. Similarly, innovative approaches, including peptide-based systems and stimuli-responsive nanoparticles, demonstrate potential to penetrate ECM barriers, deliver drugs directly to cancer cells, and minimize off-target effects, thereby improving therapeutic efficacy.

However, the dual role of the ECM as both a promoter and suppressor of tumorigenesis, its heterogeneity, and the risk of unintended consequences-such as increased invasiveness or organ dysfunction-pose significant challenges. Addressing these complexities requires continued research to identify reliable biomarkers, refine ECM-targeting strategies, and integrate these approaches with existing therapies like immunotherapy. By tackling these challenges, the ECM holds the potential to transform cancer treatment, providing novel pathways to combat metastasis, chemoresistance, and disease progression.

## Data Availability

No datasets were generated or analysed during the current study.
